# Tailored-Dose Baclofen in the Management of Alcoholism: A Retrospective Study of 144 Outpatients Followed for 3 Years in a French General Practice

**DOI:** 10.3389/fpsyt.2018.00486

**Published:** 2018-10-08

**Authors:** Juliette Pinot, Laurent Rigal, Bernard Granger, Stéphanie Sidorkiewicz, Philippe Jaury

**Affiliations:** ^1^Département de Médecine Générale, Université Paris Descartes, Sorbonne Paris Cité, Faculté de Médecine, Paris, France; ^2^Département de Médecine Générale, Université Paris Sud, Paris Saclay, Faculté de Médecine, Paris, France; ^3^Faculté de Médecine, Service de Psychiatrie et d'Addictologie Hôpital Tarnier, Hôpitaux Universitaire Paris Centre, Assistance publique—Hôpitaux de Paris, Université Paris Descartes, Sorbonne Paris Cité, Paris, France

**Keywords:** alcoholism, baclofen, retrospective study, primary care, long-term treatment

## Abstract

**Background:** More information is needed about the efficacy and safety of long-term baclofen in the treatment of alcohol use disorders. The objective of this study was to assess the effect of treatment with tailored-dose baclofen on alcohol consumption in patients with alcohol use disorders followed for 3 years after first initiating baclofen treatment.

**Methods:** This retrospective descriptive cohort included outpatients followed in a French general practice clinic for 3 years and treated with tailored-dose baclofen to reduce or eliminate alcohol consumption. At 3 years, treatment was considered successful if alcohol consumption was at or below levels defined as low-risk by the WHO (≤ 40 g/d in men and ≤ 20 g/d in women).

**Results:** The study population included 144 patients (88 men and 56 women). The participants' mean age was 46 ± 11 years and mean daily alcohol intake before treatment was 167 ± 77 grams. At the end of the study, treatment was successful for 91 (63.2%) patients. Participants' mean dose of baclofen at the end of study period was 100 ± 101 mg/d. We identified 75 (52.1%) patients for whom treatment was successful at each annual follow-up appointment: at 1, 2, and 3 years. The mean maximum dose of baclofen over follow-up of the 144 patients was 211 ± 99 mg/d (dose range: 40 mg/d to 520 mg/d).

**Conclusion:** In this study, tailored-dose baclofen appears to be an effective treatment in patients with alcohol use disorders, with sustainable effect over time (3 years). There are many adverse effects but they are consistent with those already described in the literature.

## Introduction

Baclofen, a gamma-aminobutyric acid B-receptor agonist, has been prescribed for more than 40 years for treating spasticity caused by diseases of the central nervous system, at recommended doses between 30 and 80 mg/day. It appears to be a promising candidate for treating patients with alcohol use disorders (1), by reducing or even suppressing their craving.

The first randomized placebo-controlled double-blind trial to assess the benefits of baclofen at 30 mg/day to treat alcohol dependence was published in 2002 (2). Subsequent randomized placebo-controlled studies of doses up to 60 mg/day reported contradictory results (2–8). Four of these 7 studies reported positive findings for the primary outcome.

At the same time, several case reports (9–12) and retrospective observational studies (13–15) indicated that high-dose baclofen (up to 400 mg/d) could be effective for treating alcohol dependence. One randomized placebo-controlled double-blinded trial (Baclad) assessing high-dose baclofen (up to 270 mg/day) over 6 months for treating alcohol dependence reported a significantly higher proportion of abstinence with baclofen vs. placebo (42.9% vs. 14.3%, *P* = 0.037) (16). Another randomized placebo-controlled double-blind trial of doses up to 150 mg/day over 4 months in alcohol-withdrawn patients did not find a difference in the groups from the first initiating treatment to the first relapse (17). Two randomized placebo-controlled double-blind trials of high-dose baclofen were conducted in France (18, 19). The Alpadir study did not demonstrate the efficacy of baclofen at the target dose of 180 mg/day in maintaining abstinence over 6 months (19). The Bacloville study reported a significantly higher proportion of patients with a low risk alcohol consumption (WHO) or abstinent in baclofen group (doses up to 300 mg/day) vs. placebo group after 1 year (18).

The maximal duration of the follow-up in these randomized placebo-controlled double-blind trials was 1 year and the longest retrospective study was for 2 years. Hence, we need information on long-term baclofen treatment. Indeed, studies are needed on whether patients who became abstinent or managed to control their alcohol consumption with baclofen could stop or reduce the treatment while remaining abstinent or controlling their consumption.

Here we present the retrospective clinical experience of a general practitioner (PJ) who has prescribed tailored-dose baclofen since 2008 for patients at high risk for alcoholism as defined by the World Health Organization (WHO; >40 g/day for women and >60 g/day for men). The primary objective of this study was to assess effectiveness of tailored-dose baclofen for alcohol consumption in patients with alcohol use disorders who were followed over 3 years after first initiating baclofen. Secondary objectives were to analyse the dose of baclofen prescribed during the 3 years of follow-up and tolerance of the treatment and to explore patient characteristics associated with low-risk alcohol consumption.

## Methods

### Study design

This was a retrospective study among patients of a French general practitioner (PJ) trained in addictology. Patients were eligible for the study if they (1) were at least 18 years old, (2) drank at a high-risk level according to the WHO (20), (3) first began taking baclofen before December 31, 2011, and (4) were willing to be followed up for more than 3 months to assure stability of patient care.

Eligible patients were identified from an exhaustive list of patients who received a baclofen prescription that the general practitioner (PJ) maintained. For each patient, data were collected retrospectively from their medical file by one independent investigator (JP). If necessary, data collection was completed by asking the patient questions during a consultation with the general practitioner or by telephone with the investigator.

### General practitioner follow-up and prescription

Patients did not necessarily stop drinking alcohol before beginning baclofen treatment. They could drink alcohol with the treatment. Baclofen was prescribed at progressively increasing doses according to the standard of care at the time. This practice was later published in prescription guidelines (21) without any pre-set limit up to the dose that allowed patients to control their alcohol consumption or even become indifferent to it. The objective for this treatment was harm reduction (as proposed by European Medicines Agency, 2010, and confirmed by recommendations from the US Food and Drug Administration, 2015, and UK Chief Medical Officers' Low Risk Drinking Guidelines, 2016) (22). Each patient received comprehensive care according to NICE guidelines (23), with or without the additional prescription of psychotropic drugs. Each patient received written and oral information about the treatment (modality of prescription, follow-up, main side effects, potential risks of combining higher doses of baclofen and high volumes of alcohol importance of stopping treatment progressively).

### Data collection

The following data were collected:

- social and demographic data: age, sex, marital status, work status
- history of treatment for management of alcoholism: episodes of detoxification, participation in discussion groups, drug treatment (e.g., acamprosate, naltrexone, or disulfiram)
- psychiatric disorders: depression, bipolar, anxiety, or borderline personality disorders
- history of use of other toxic substances: tobacco, cannabis, heroin, cocaine
- history of eating disorders
- history of insomnia
- at treatment initiation and at 1, 2, and 3 years follow-up visits: reported alcohol consumption (in grams per day), daily baclofen dose (in milligrams), consumption of other toxic substances (tobacco, cannabis, heroin, cocaine), a list and dose of psychotropic drugs (anxiolytics, hypnotics, antidepressants, antipsychotics, and mood stabilizers), a list and dose of psychotropic drugs used as substitutes for opiates
- maximum dose of baclofen taken during follow-up.
- tolerability of baclofen (reported by the patient at each consultation). We focused on the sides effects reported by patients during the first year of follow-up given that it corresponds to the phase of baclofen initiation and titration. After titration, we only reported serious adverse effects (hospitalizations and deaths).

### Outcome

Treatment was considered successful if patients were either abstinent or drinking at a low-risk level as defined by the WHO (≤ 40 g/day for men and ≤ 20 g/day for women) at 3 years after beginning the treatment even if no longer taking baclofen.

### Data analyses

We analyzed data for patients who were eligible for the study and for whom data on alcohol consumption was provided during the 3 years follow-up. Descriptive analyses were used for alcohol consumption defined according to the WHO risk categories and baclofen dosage during follow-up. Pearson's correlation analysis was used to determine correlation between the maximum baclofen dose used for efficacy and patient characteristics as well as daily alcohol intake before starting baclofen. We attempted to identify factors associated with treatment success (baseline characteristics of patients associated with successful treatment at 3 years in univariate with *p* < 0.20 were considered in multivariate logistic regression). We described any adverse effects and compared patients lost to follow-up before 3 years to those who completed follow-up. Chi-square or Fisher's exact test was used as appropriate to analyze categorical variables and Student *t*-test for quantitative variables. Statistical analyses involved use of STATA v12.0 and R 3.2.5. *P* < 0.05 was considered statistically significant.

### Ethical aspects

All patients included were informed of the study objective and provided informed consent during a consultation with the general practitioner or by telephone with the investigator. This study was not reviewed by a research ethics committee because as a retrospective study, it was not at this time within the scope of the French statutes regulating biomedical research.

## Results

### Descriptive characteristics

We identified 219 eligible patients who initiated baclofen treatment before December 31, 2011: 75 were lost to follow-up, including 6 who died, resulting in a final cohort of 144 (65.8%) for analysis.

The mean (SD) age at inclusion was 46 (11) years and 88 (61.1%) were men (Table 1). The mean (SD) quantity of alcohol consumed daily at baclofen initiation was 167 (77) g. Overall, 103 patients (71.5%) previously received treatment for alcohol use disorder (drugs or detoxication or discussion groups). At baclofen initiation, 120 patients (83.3%) had a psychiatric disorder [Diagnostic and Statistical Manual of Mental Disorders, 4th [DSM-IV] criteria]. Before beginning baclofen treatment, 69.4% were taking at least one psychotropic drug.

**Table 1:** Baseline characteristics of patients who completed and did not complete follow-up at 3 years (lost to follow-up or died).

	**Completed follow-up (*N* = 144)**	**Did not complete follow-up (*N* = 75)**	***p***
Male	88 (61.1)	46 (61.3)	0.974
Age (years), mean (SD)	46.43 (11.00)	46.51 (11.16)	0.962
Living with a partner	83 (57.8)	41 (55.4)	0.742
Employed	85 (59.4)	44 (59.5)	0.998
Had tried a drug approved for relapse prevention	91 (63.2)	46 (61.3)	0.787
- acamprosate	71 (49.3)	27 (36.0)	0.060
- naltrexone	62 (43.1)	28 (37.3)	0.414
- disulfiram	21 (14.6)	11 (14.7)	0.987
Had undergone detoxification	47 (32.6)	29 (38.7)	0.374
Had participated in discussion groups	38 (26.4)	14 (18.9)	0.220
Previously treated for alcoholism (drugs or detoxications or discussion groups)	103 (71.5)	54 (72.0)	0.941
Quantity of alcohol consumed daily at inclusion (g), mean (SD)	167 (77)	159 (119)	0.582
Psychiatric disorders (clinician assessment)	120 (83.3)	57 (76.0)	0.191
- anxiety	96 (66.7)	44 (58.7)	0.242
- depression	90 (62.5)	44 (58.7)	0.581
- borderline personality	23 (16.0)	5 (6.7)	0.056
- psychosis	5 (3.5)	3 (4.0)	1.00
- bipolar disorders	7 (4.9)	4 (5.3)	1.00
Other addictions:			
- smoking	109 (75.7)	44 (58.7)	**0.009**
- cannabis	29 (20.1)	13 (17.3)	0.617
- heroin	1 (0.7)	0 (0)	1.00
- cocaine	6 (4.2)	2 (2.7)	0.718
History of eating disorders	7 (4.9)	3 (4.1)	1.00
Treatment at baseline: psychotropic drugs	100 (69.4)	40 (53.3)	**0.018**
- anxiolytics and hypnotics	84 (58.3)	35 (46.7)	0.133
- antidepressants	60 (41.7)	17 (22.7)	**0.005**
- antipsychotics	13 (9.0)	6 (8.0)	0.798
- mood stabilizers	5 (3.5)	2 (2.7)	1.000
Treatment at baseline: opiate substitutes (buprenorphine and methadone)	19 (13.2)	3 (4.0)	**0.034**

*Data are n (%) unless indicated. Bold values correspond to values where p < 0.05*.

### Alcohol consumption and baclofen dose

At 3-year follow-up, treatment was successful for 91 patients (63.2%): 61 abstinent (42.4%) and 30 low-risk drinkers (20.8%) according to the WHO classification. Had all the patients lost to follow-up analyzed and classified as failures, the success rate at 3 years would have been 41.6%.

**Figure 1:**
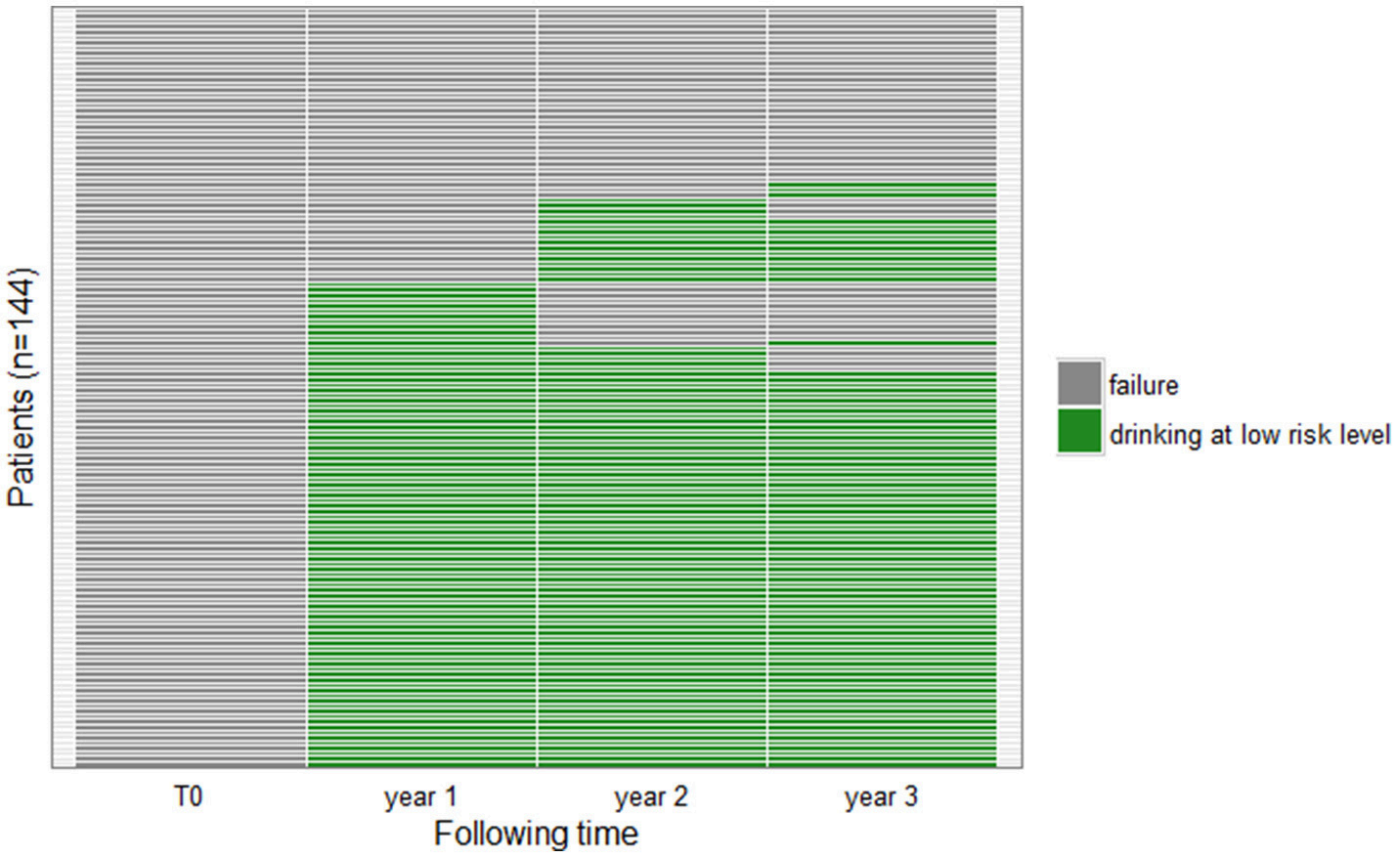
Patient outcomes during follow-up. Each row corresponds to one patient. The colors correspond to drinking levels for patients at 3 follow-up visits (year 1, year 2, year 3): green: abstinent or low-risk drinkers; gray: failure.

For 75 patients (52.1%), treatment was successful at each annual follow-up visit, at 1, 2, and 3 years (Figure 1).

At 3 years, the mean (SD) daily dose of baclofen was higher for patients with than without successful treatment (100 [101] vs. 58 [102] mg/day, *P* = 0.017). In all, 29 patients with successful treatment were no longer taking baclofen at 3 years (i.e., 20.1% of all patients and 31.9% with successful treatment).

**Figure 2:**
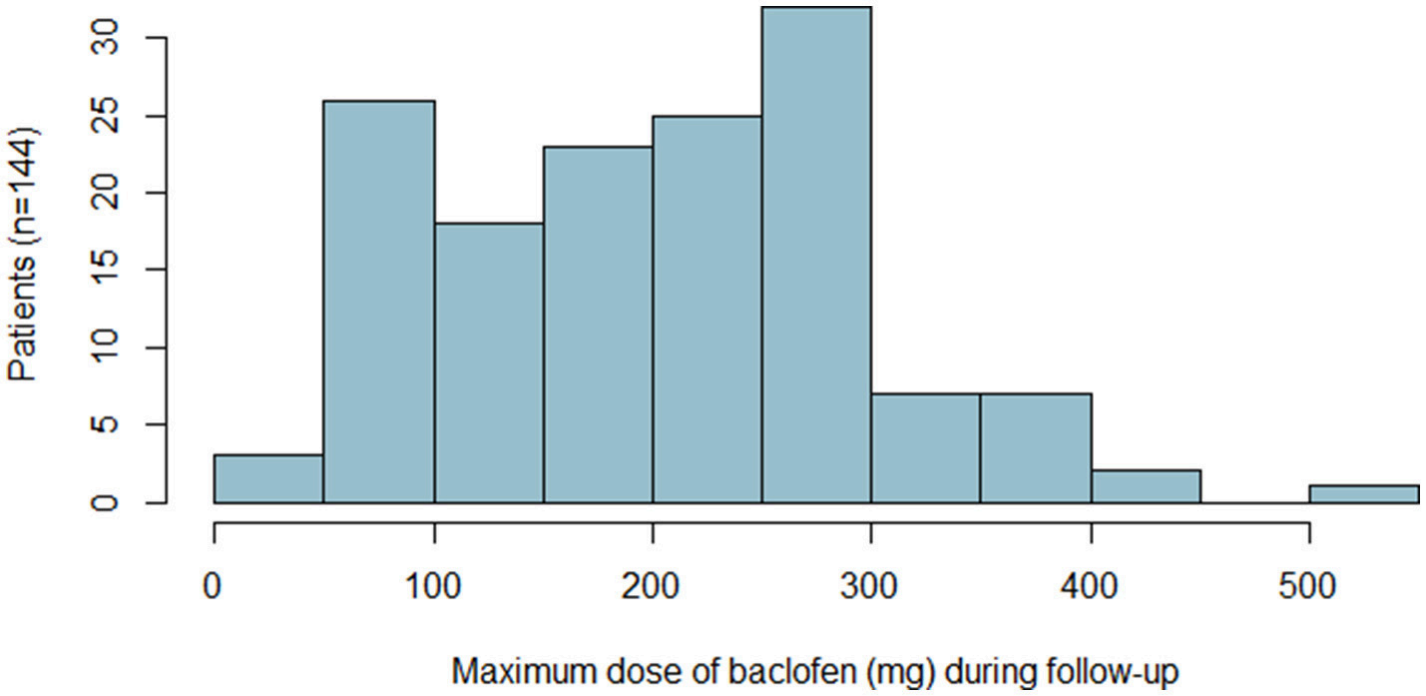
Maximum dose of baclofen taken by patients with the number of patients.

The mean (SD) maximum dose of baclofen during follow-up was 211 (99) mg/day (range: 40–520) and the median was 210 mg/day (first quartile: 120, third quartile: 300) (Figure 2). The mean (SD) maximum dose prescribed did not differ significantly between patients with and without successful treatment (219 [98] vs. 194 [100] mg/day, *P* = 0.137).

We found a significant but low correlation between alcohol consumption at inclusion and maximum baclofen dose (*r* = 0.2607, *P* = 0.0017).

At 3 years, in univariate and multivariate analysis the two outcome groups did not differ significantly in any baseline characteristics (social and demographic characteristics, alcohol intake, previous treatment for alcoholism, other addictions, psychiatric disorders, or psychotropic medication).

For the 75 patients with successful treatment at each annual follow-up visit, the mean (SD) maximum dose of baclofen during follow-up was 220 (100) mg/day (range: 60–520) (Table 2) and the median was 240 mg/day (first quartile: 150, third quartile: 300); 22 could stop baclofen and the other 53 could reduce the maximum baclofen dose by an average of 35%.

**Table 2:** Baclofen dose during follow-up for patients with successful treatment at each yearly follow-up visit (*n* = 75).

	**1 year**	**2 years**	**3 years**
Patients no longer taking baclofen—n (%)	13 (17.3%)	18 (24.0%)	22 (29.3%)
Patients taking baclofen—*n* (%)	62 (82.7%)	57 (76.0%)	53 (70.7%)
Current baclofen dose (mg/d), mean (SD)	172 (100)	142 (95)	143 (91)

### Adverse effects

During the first year of follow-up (including the phase of baclofen initiation and titration) 16 patients (11.2%) reported no adverse effects. The most frequently adverse effects, reported by 128 (88.8%) patients during year 1, included drowsiness (48.6%), asthenia (37.5%), insomnia (28.5%), vertigo (20.8%), nausea (18.8%), headaches (16.7%), sudden fatigue (12.5%), concentration disorders (11.8%), sweating (11.8%), hypomania (11.8%), and memory disorders (10.4%).

During follow-up, ten serious adverse effects occurred: four patients were hospitalized and six died.

Three patients were hospitalized for confusion: one patient had a baclofen overdose and two patients did not follow the general practitioner's prescriptions and recommendations (they specifically stopped baclofen abruptly and then resumed it at a high-dose without titration; moreover, one of the two patients also consumed a high dose of alcohol at the same time). One patient was hospitalized for encephalopathy: this patient abruptly stopped and then resumed a high dose of baclofen without titration. These hospitalizations led to the discontinuation of baclofen for all four patients. One of these patients was classified as a success at each annual follow-up visit at 1, 2, and 3 years. For two patients, outcome was successful only at 1 year. And the fourth patient was lost to follow-up before 1 year.

Concerning the six patient who died during follow-up, the general practitioner considered that they were not related to baclofen. Three patients with long-standing psychiatric disorders committed suicide (two had not taken baclofen for more than 6 months), two overdosed with heroin (one had not taken baclofen for 3 months), and one patient died during an alcohol coma.

### Comparison of patients completing and not completing follow-up (lost to follow-up or died)

Patients completing follow-up were more frequently current smokers (75.7 vs. 58.7%, *P* = 0.009) and taking antidepressants (41.7 vs. 22.7%, *P* = 0.005) or opiate replacements (13.2 vs. 4.0%, *P* = 0.034). The two groups were comparable in all other characteristics (Table 1).

## Discussion

This study aimed to assess the efficacy of tailored-dose baclofen for alcohol consumption in 144 patients with alcohol use disorder who were followed over 3 years after first initiating baclofen. At the end of the follow-up, treatment was successful for 63.2% of patients—they were abstinent or drinking at a low-risk level—and their mean (SD) dose of baclofen was 100 (101) mg/day. Our study is the first with a follow-up of 3 years and thus could assess the efficacy and the safety of the treatment over a longer term than previous studies.

We did not find any association between patient characteristics at inclusion and their alcohol consumption reported in the WHO risk categories at 3 years. Nor did we identify any particular patient profile or characteristics associated with a good response to baclofen.

Moreover, we found a significant but low correlation between the maximum dose of baclofen and alcohol consumption at inclusion, as previously reported by de Beaurepaire (14) and Shukla et al. (24). This result suggests that the higher the initial alcohol intake, the higher the baclofen dose needed to control it.

One limitation is that our study was a single-center with a single prescriber retrospective cohort, so the generalizability of our findings may be limited, and our results are prone to biases inherent of this type of design. The alcohol use disorder requires a long-term care, this is the reason why patients (125) with a follow-up of < 3 months were excluded because alcohol use disorder requires a long-term care. Among this group, some (46) were seen only once and probably never began the baclofen treatment. Others were seen for a short period for an opinion or to begin the treatment before being followed up by a doctor closer to their home. The other patients excluded were probably not sufficiently motivated to undergo treatment for their drinking problem. For those patients with insufficient motivation to undergo treatment, it is important to develop specific alcohol care packages.

At 3 years, 32.4% of the patients had been lost to follow-up. The investigator (JP) sought to contact the 116 patients no longer seen by the general practitioner at 3 years; data were completed for 47 patients. Among the patients lost to follow-up, some had changed their telephone number (*n* = 21, 30.4%), some did not want to respond to questions on the telephone (*n* = 9, 13.0%), and others never responded to calls (*n* = 39, 56.5%). Some patients had requested a prescription for high-dose baclofen before the doctor suggested it. These patients had a positive view of the treatment in advance, which might have accentuated the placebo effect. Another limitation is linked to the retrospective design of the study. We were unable to corroborate the alcohol consumption reported by patients by biomarker testing or questioning family or friends. Therefore, the effect of baclofen on alcohol consumption we found may be overestimated. Some of the observed effect may be associated with the intervention by the physician (motivational interview and psychological support) or family and friends. All patients were offered the same follow-up and treatment. Nonetheless, the management of alcoholism is complex and cannot be summarized by the simple prescription of a medication.

One of the main strengths of the study is the large number of patients and the duration of follow-up: 144 patients at 3 years. The other observational studies of baclofen prescription in alcoholism analyzed fewer patients for shorter periods. Moreover, our study had a follow-up at 1 and 2 years. Thus, 52.1% of patients had successful outcomes at each of the 1, 2, and 3 years visits. The effect of baclofen on alcohol consumption appears to be sustainable.

In our study, the baclofen prescription dose was progressively increased, without any pre-set limit: it ranged from 40 to 520 mg/day. The mean (SD) maximum dose prescribed was 211 (99) mg/day, which was higher in our study than the mean (SD) dose prescribed in the studies by Rigal et al. and de Beaurepaire: 145 (75) mg/day (range: 30–400), 159 (87) mg/day (range: 30–400), and 147 mg/day (range: 20–330) (13–15). In two randomized placebo-controlled double-blind trials examining the highest doses of baclofen before our study, the baclofen doses prescribed ranged from 30 to 270 mg/day for one (mean [SD] maximum baclofen dose was 180 [87] mg/day) (16) and from 15 to 300 mg/day for the second (18). Hence, the higher mean baclofen doses in our study as compared with the literature provides further information about the tolerability and safety of high-dose baclofen prescription. The adverse events we observed in our study suggest that future prescribing physicians need to be carefully informed and trained about potential severe events.

One of the original aspects of this study [as for Rigal et al. (13, 15)] is that it included patients who were not necessarily alcohol-dependent according to DSM-IV criteria. All patients had alcohol use disorders and wanted medical assistance.

Baclofen prescription was pragmatic. Patients with psychiatric disorders or using psychotropic medication or illegal drugs were included and maintained their usual treatments. Psychiatric disorders and the use of psychotropic medication were often exclusion criteria for previous randomized double-blind placebo-controlled studies of baclofen used to treat alcohol dependence (3–5, 8, 16). Some exceptions were Beraha et al. including patients with depression, anxiety or bipolar disorder (17); the Alpadir study including patients using an antidepressant at a stable dose for 2 months or anxiolytics such as diazepam or oxazepam and excluding only patients with severe psychiatric disease (19); and the Bacloville study excluding only patients with severe psychiatric disorders that could compromise their participation in the study (18).

## Conclusion

Tailored-dose baclofen seems an effective treatment for patients with alcohol use disorders, with sustainable effect over time (3 years). We found many adverse effects, but they are consistent with those described in the literature.

## Author contributions

All patients were from the general practitioner's practice (PJ). JP and LR participated in the conception and design of the study. JP collected the data. JP and SS performed the statistical analysis. All authors interpreted the results. JP drafted the manuscript and all authors revised the manuscript critically for important intellectual content. All authors read and approved the final version of the manuscript.

### Conflict of interest statement

PJ received consultancy fees from Polpharma. The remaining authors declare that the research was conducted in the absence of any commercial or financial relationships that could be construed as a potential conflict of interest.
